# Value-based health care frameworks for the health technology assessments of “omics” technologies: an international survey

**DOI:** 10.1017/S0266462325103279

**Published:** 2025-11-28

**Authors:** Tommaso Osti, Abdelrahman Taha, Eva Reviriego-Rodrigo, Roberta Pastorino, Stefania Boccia, Iñaki Gutierrez-Ibarluzea

**Affiliations:** 1 Department of Life Sciences and Public Health, Section of Hygiene, Universita Cattolica del Sacro Cuore, Rome, Italy; 2 Basque Office for Health Technology Assessment, BIOEF, Basque Foundation for Health Innovation and Research, Barakaldo, Spain; 3 Departments of Woman and Child Health and Public Health, Fondazione Policlinico Universitario Agostino Gemelli IRCCS, Rome, Italy; 4 Health Research, Innovation and Evaluation Directorate, Ministry for Health, Basque Government, Bilbao, Spain

**Keywords:** health technology assessment, genomics, precision medicine, personalised medicine, assessment framework, value-based healthcare

## Abstract

**Background:**

Despite omics technologies gaining traction in clinical settings, particularly in oncology, challenges persist in their widespread adoption due to the pre-requisite robust evidence supporting efficacy and cost-effectiveness. This study aims to explore the experiences of organizations working in the health technology assessment (HTA) field in evaluating omics technologies, with a particular focus on the adoption and application of specific assessment frameworks.

**Methods:**

We conducted a global survey to gather insights into current practices and frameworks used in HTA evaluations of omics technologies.

**Results:**

We gathered responses from thirty-nine participants representing organizations across twenty-nine countries and five continents. Among them, 51 percent (n = 20) reported experience in evaluating omics technologies, including multi-omics tests for early disease detection, biomarker-based cancer diagnostics, and advanced genomic sequencing techniques. Only three organizations employed specific assessment frameworks: the *Adelaide Health Technology Assessment Agency* in Australia, the *Netherlands Cancer Institute*, and the *Andalusian HTA Agency* in Spain. These frameworks address key evaluation aspects such as analytical and clinical validity, clinical and personal utility, organizational impact, and ethical, legal, and social implications of omics technologies.

**Discussion:**

Despite their relevance, the limited adoption of tailored frameworks highlights the need for more structured and context-specific approaches to facilitate the integration of omics technologies into healthcare systems. Collaborative efforts among stakeholders, including patients, healthcare providers, policymakers, and industry representatives, are crucial for devising robust evaluation strategies addressing the complexities of omics technologies comprehensively.

## Introduction

Technology has driven the emergence of precision medicine, marking a paradigm shift in healthcare. By integrating individual genetic, environmental, and lifestyle factors, precision medicine enables personalized health promotion, disease prevention, diagnosis, and treatment (1). This transformative approach has catalyzed the development of cutting-edge technologies aimed at enhancing our understanding of individual biological variables. Omics sciences, comprising disciplines such as metabolomics, proteomics, epigenetics, and transcriptomics, have flourished, providing a holistic understanding of biological systems, paving the way for tailored healthcare solutions (2;3). Over the years, omics technologies have been integrated into clinical practice, particularly in oncology. Genomic tests, for instance, have facilitated the identification of predisposing hereditary syndromes (e.g., breast and ovarian cancer), consequently allowing the implementation of targeted screening and risk reduction strategies based on genomic data (4). Additionally, multi-omics tools have provided insights into disease mechanisms and therapeutic responses, enhancing treatment precision (5). Despite these advancements, the widespread adoption of these technologies in clinical practice remains limited, primarily due to challenges in the generation of robust evidence supporting their efficacy and cost-effectiveness (6;7). In this sense, health technology assessment (HTA) is considered a central tool for evaluating evidence to support the potential implementation of novel tools. HTA considers the clinical, economic, organizational, and societal implications of healthcare innovations, including omics technologies (8). This methodology, leveraging the concept of value-based healthcare (VBHC), can promote a multidimensional concept of value that integrates patient-centered outcomes, system sustainability, and equity considerations (9). HTA involves a systematic and multidisciplinary evaluation of health technologies, balancing both their direct clinical benefits and broader societal impacts (10), but while HTA frameworks are successfully applied to assess other healthcare interventions, the complex nature of omics technologies poses unique challenges. Each tool is composed of a set of a biomarker, a diagnostic assay, and a subsequent therapeutic intervention. The validation process for these components therefore requires that each of these elements is evaluated from multiple perspectives, thus creating multiple layers of complexity in generating the necessary evidence (11;12).

One major consequence of this complexity is the reliance on non-traditional study designs. While randomized controlled trials (RCTs) are the gold standard for evaluating medical interventions, they often fall short in assessing omics technologies. As highlighted by a Food and Drug Administration analysis (13) RCTs typically rely on standardized protocols and large sample sizes to measure treatment effects across a population; however, omics technologies emphasize personalized and tailored approaches to healthcare, necessitating interventions that are specifically tailored to the individual characteristics of patients. This inherent variability makes it difficult to design RCTs that adequately capture the diverse effects of omics interventions across different patient populations (14). Furthermore, the ethical, legal, organizational, and social considerations (ELSI) associated with omics technologies present additional challenges in the HTA process (15). These elements include data privacy, informed consent, and equitable access to healthcare (16). Moreover, issues on equity and access arise concerning the availability and affordability of omics technologies, particularly in underserved or socio-economically deprived communities (17).

This results in a context characterized, on the one hand, by the clear necessity of employing HTA methodologies to appropriately evaluate the potential implementation of omics technologies within healthcare systems, and on the other hand, by the need to tailor these methodologies to their distinctive characteristics. This scenario unfolds against a backdrop in which existing scientific literature highlights a gap in clearly defining the assessment tools currently utilized by national HTA organizations for these evaluations (18). Without a clear overview of the current landscape, it is challenging to understand the state of the art or to adopt novel assessment frameworks capable of effectively addressing the evaluative demands specific to omics sciences.

This study aims to directly address this literature gap by exploring how different regional and national HTA organizations practically approach the evaluation of omics technologies. Specifically, our objectives include defining an overview of existing HTA frameworks and methodologies in use, identifying perceived challenges and potential solutions as reported by institutions themselves, and highlighting convergence and divergence points across organizations. By adopting this broader perspective, we aim to foster the development of more robust, coherent, and adaptive HTA strategies. Ultimately, the goal is to support informed decision-making processes that responsibly integrate omics technologies into clinical practice and health systems, optimizing their potential to advance precision medicine.

## Methods

We developed and administered a survey to representatives from organizations working in the HTA field to gather insights on the way they perform assessments of omics technologies, the use of specific evaluation frameworks, and their characteristics.

### Survey development and content

The survey was designed, reviewed, and approved by the research team. The first section of the survey collected background information, including the country of the responding organization and the primary focus of its activities. Subsequent questions focused on the agency’s experience in evaluating omics technologies and its use of specific assessment frameworks. The complete survey is available in Supplementary Materials (Supplementary 1). The survey was distributed via the online platform *Encuesta fácil* (www.encuestafacil.com).

### Survey population and recruitment

To ensure broad geographical representation of organizations as recipients of the survey, a mapping exercise was conducted to identify organizations operating in the field of HTA globally. The mapping process leveraged the *International Network of Agencies for Health Technology Assessment (INAHTA)* website (https://www.inahta.org/members/) and other official repositories, leading to the identification of seventy-five organizations, including forty-five in Europe and the UK and thirty in other regions worldwide. Additionally, the research team supplemented this list with relevant contacts from their professional networks. Each identified organization with a documented record of producing HTA reports or contributing to HTA evaluations was contacted by e-mail and invited to nominate a senior analyst or coordinator with direct experience in technology assessment to complete the survey. To reduce the potential impact of non-response bias, multiple reminder e-mails were sent after the initial invitation, and respondents were explicitly encouraged to provide answers reflecting the practices of their institutions rather than individual views. Although the risk of non-response bias was not formally assessed, the recruitment strategy was designed to maximize diversity in geographical coverage and institutional profiles. Data collection was kept open until heterogeneity was reached, ensuring that the final sample reflected an acceptable balance in terms of geographic distribution.

Data collection occurred from December 2023 to March 2024. All responses were treated as confidential. While participants could choose to remain anonymous, those who wished to receive study outcomes provided their email addresses, which were stored separately from the main dataset to maintain confidentiality.

### Statistical analysis

Descriptive statistics, including absolute frequencies and percentages, were generated using *Encuesta fácil* (www.encuestafacil.com). Responses to open-text questions were independently coded by two researchers and then conflicts were resolved by a third researcher to reach consensus. Results were thematically analyzed and presented in a descriptive format (19).

## Results

**Figure 1.**
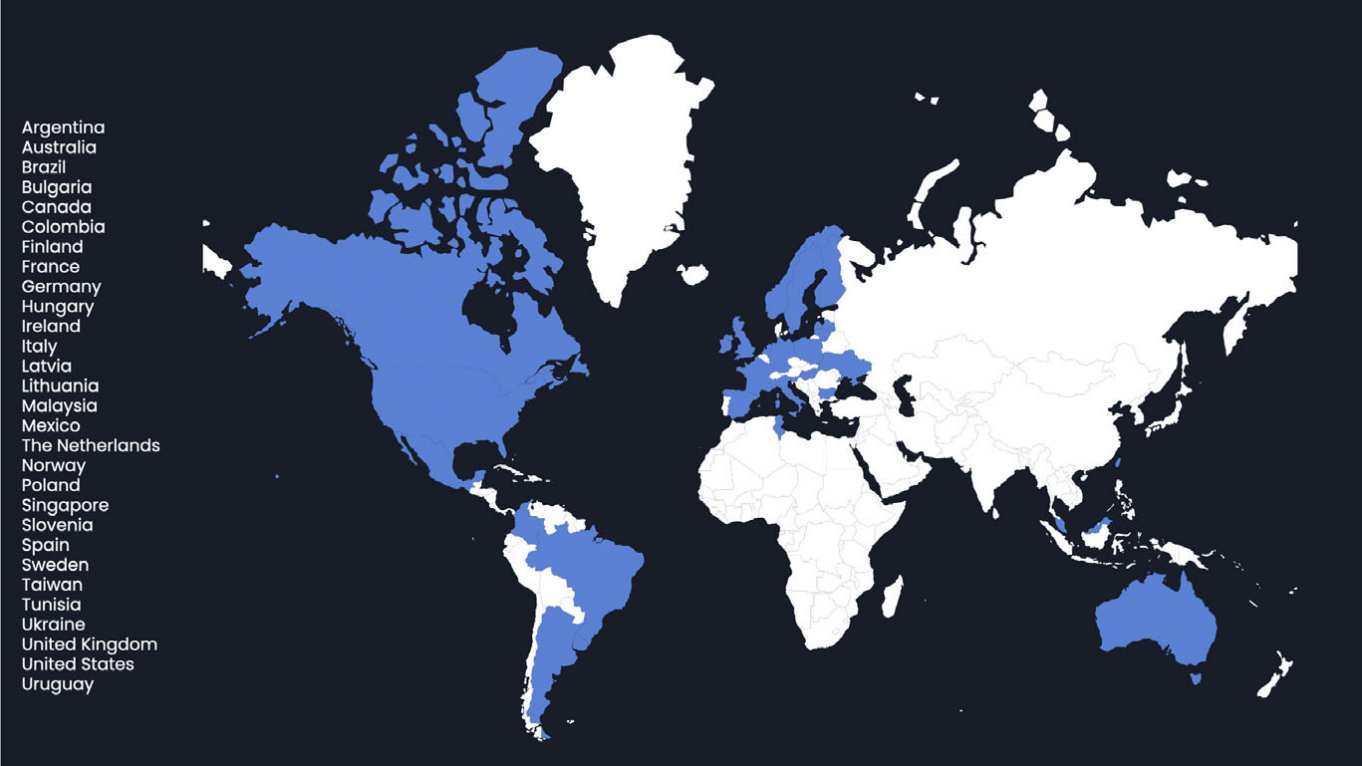
Geographical distribution of survey respondents. The map highlights the twenty-nine countries (in blue) from which thirty-nine organizations participated in the survey on HTA frameworks for omics technologies. The list on the left specifies all the countries represented among respondents.

The survey was sent to seventy-five agencies and organizations, thirty-nine respondents participated in the survey, yielding a response rate of 52 percent, each representing a distinct organization operating in the field of HTA. These respondents hailed from twenty-nine countries across five continents. Figure 1 illustrates the geographical distribution of respondents.

**Figure 2.**
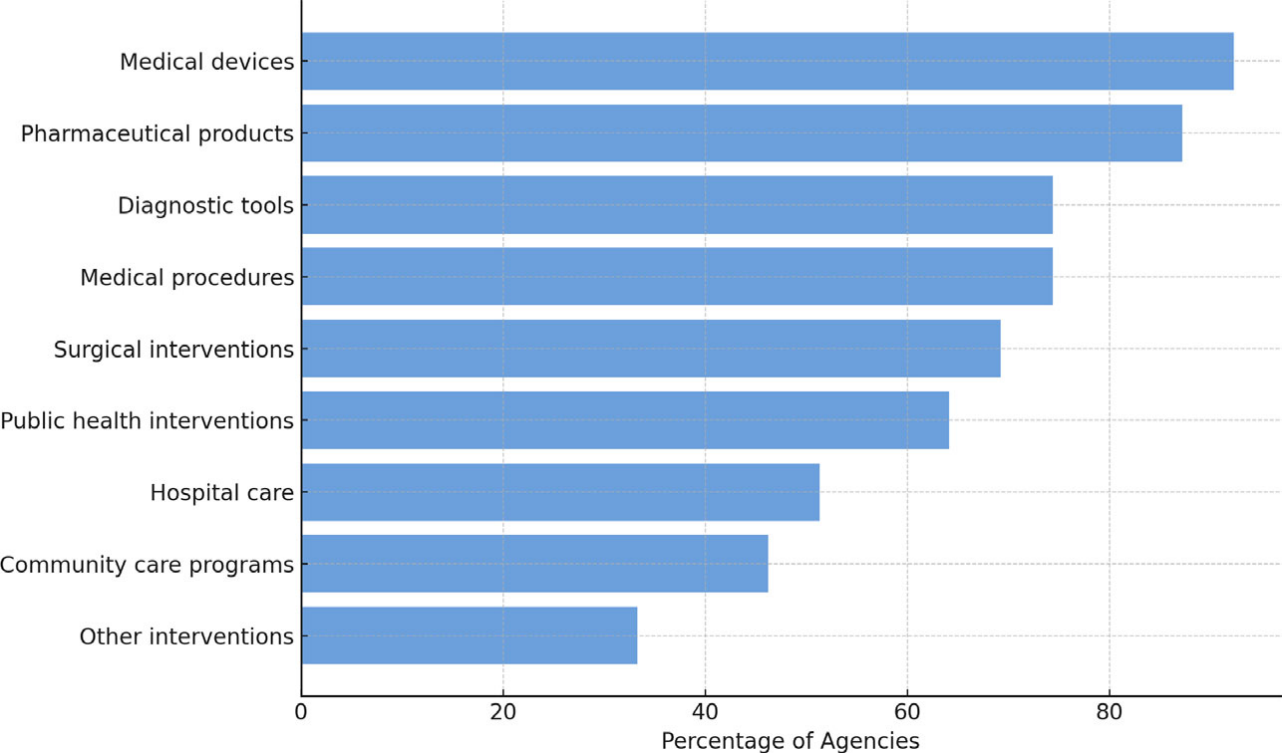
Types of technologies evaluated by HTA organizations. The chart shows the percentage of surveyed organizations reporting evaluations across different categories of health technologies. Medical devices and pharmaceutical products are the most frequently assessed, followed by diagnostic tools and medical procedures.

Regarding the primary areas of focus of the agencies and organizations, thirty-four (87.2 percent) assessed pharmaceutical products, while medical devices were evaluated by thirty-six (92.3 percent). Twenty-nine respondents (74.4 percent) examined diagnostic tools, twenty-seven (69.2 percent) surgical interventions, and twenty-nine covered (74.4 percent) medical procedures. Furthermore, twenty organizations (51.3 percent) evaluated hospital care, community care programs were addressed by eighteen (46.2 percent), and twenty-five (64.1 percent) reviewed public health interventions. Thirteen (33.3 percent) respondents reported evaluating other specific interventions, including consumable medical equipment, dentistry interventions, psychotherapeutic interventions, and non-medicine health technologies. Figure 2 provides an overview of the distribution of the respondents across these domains (20).

Twenty (51.3 percent) organizations reported experience in evaluating omics technologies, heterogeneously distributed within the categories “Pharmaceuticals products” and “Diagnostic tools.” The specific types of technologies assessed included:

- Multi-omics tests for early diagnosis of chronic conditions;
- Gene therapy products;
- Biomarker-based tests to guide cancer therapy decisions;
- Comprehensive genomic profiling using high-throughput next-generation sequencing (NGS) across multiple fields;
- Liquid biopsy techniques for detecting and analyzing circulating tumor DNA (ctDNA);
- Whole-genome sequencing (WGS) applied to various conditions;
- Genomic sequencing and transcriptomic analysis for diagnosing developmental disabilities, congenital anomalies, and neurological disorders.
- Comparative genomic hybridization arrays, microarray, DNA sequencing (Sanger sequencing and NGS), and polymerase chain reaction for diagnostics and research applications.

Out of twenty organizations with direct experience in HTA for Omics technologies, three reported using a specific methodological framework for their assessment. These are the *Adelaide Health Technology Assessment* Agency (AHTA) in Australia, the *Netherlands Cancer Institute*, a comprehensive cancer center where HTA are performed in The Netherlands, and the *Health Technology Assessment Area-AETSA, Andalusian Public Foundation Progress and Health-FPS.* The characteristics of the three evaluation frameworks are reported below and summarized in Table 1.

**Table 1.** Overview of the included frameworks

Framework	Key characteristics	Setting	Limitations	VBHC elements
MSAC (Medical Services Advisory Committee)	Structured approach emphasizing methodological rigor, transparent procedures, incorporation of economic evaluations, consumer perspectives, and ethical, legal, social, organizational considerations. Adaptable to omics technologies.	Federal-level funding decisions for outpatient and private hospital services	Challenges in evidence sufficiency and interpreting omics-specific results.	Incorporates *equity* and patient-centered outcomes (e.g. “value of knowing”), systematic cost-utility analyses for *technical value*, and budget impact assessments for *system sustainability.*
CTA (Constructive Technology Assessment)	Proactive, anticipatory approach focusing on early-stage assessment, active stakeholder engagement, multidimensional analysis including technical, economic, ethical, social, regulatory, and organizational dimensions.	Primarily adopted in research contexts	Challenges in integrating qualitative dimensions beyond cost-effectiveness, lack of standardized methodologies.	Strong focus on *societal* and *personal value* through stakeholder and patient involvement, ethics, and equity; anticipatory management supports *long-term sustainability*, though less emphasis on standardized PROMs or cost-effectiveness.
**AETSA (Andalusian Health Technology Assessment Agency)**	Specialized, structured approach tailored explicitly to omics technologies, addressing analytical validity, clinical validity, clinical utility, personal utility, organizational aspects, and bioinformatics. Strong emphasis on patient-centered outcomes.	Regional reimbursement decision-making (still theoretical)	Perceived complexity due to comprehensiveness; not yet validated in practice.	Explicitly incorporates *personal utility* and patient-reported outcomes, addresses *equity* and allocative value, includes feasibility and cost-effectiveness for *technical and system sustainability*, plus ELSI for *societal value.*

## MSAC framework

The 2021 *Guidelines for Preparing Assessment for the Medical Services Advisory Committee (MSAC)* (21) developed by the *Adelaide Health Technology Assessment Agency* in Australia, constitute a well-established, structured framework routinely adopted for evaluating diagnostics, genetics, and genomic tests within the Australian public funding context. MSAC assessments primarily support federal-level funding decisions for outpatient and private hospital services included in the Medicare Benefits Schedule. Services provided in public hospitals are often appraised using an existing MSAC HTA as the basis or an evaluation may be commissioned separately at the state or local level, using distinct processes. Developed and refined over nearly two decades, these guidelines formalize several procedural elements, explicitly mandating components such as the PICO framework, economic evaluations (particularly cost-utility analyses), financial impact assessments, systematic integration of consumer perspectives (including the “value of knowing”), and structured application of the GRADE methodology. The MSAC framework uniquely emphasizes standardized methodological rigor, transparent procedural steps, and the formal incorporation of ethical, legal, social, organizational, and environmental considerations relevant to the Australian healthcare system. Although the MSAC guidelines were not originally designed specifically for omics technologies, they have demonstrated considerable adaptability and have been successfully applied to numerous omics-based assessments. Examples include genetic profiling testing for breast cancer (22–24), genetic testing for childhood hearing impairment (25), genetic testing for non-small-cell lung cancer (26), genome-wide microarray testing for multiple myeloma and chronic lymphocytic leukemia (27), genomic testing for diagnosing heritable cardiomyopathies (28), and WGS of antimicrobial-resistant pathogens (29). Despite these successes, evidence sufficiency, interpretability of omics-specific results, and comprehensive reporting remain key challenges highlighted in the survey, opening opportunities for further methodological refinement and framework customization to better address the distinctive characteristics inherent in omics evaluations. From a VBHC perspective, the MSAC framework integrates several core elements by combining rigorous cost-utility analyses with explicit consideration of equity and patient-centered outcomes. Notably, it embeds the concept of the *“value of knowing,”* acknowledging benefits such as diagnostic certainty and improved decision-making for patients and families, even when direct health gains are limited. This reflects both the *personal* and *societal* value pillars of VBHC, while sustainability is addressed through systematic budget impact analyses and modelling of long-term resource use.

## CTA framework

The *constructive technology assessment* (CTA) *in healthcare* (30), used by institutions such as the *Netherlands Cancer Institute*, represents a proactive and integrative approach to evaluate emerging technologies, including those in the omics sciences. As the Netherlands Cancer Institute is a research institute and not an HTA agency, the CTA framework is mostly used in research, rather than national reimbursement decision making. Initially developed outside healthcare contexts in the 1980s, CTA has since been adapted for the systematic assessment of healthcare technologies from design and development stages through to implementation. Characteristically, CTA is an evaluation framework that emphasizes early-stage assessment, scenario drafting, and active stakeholder engagement, enabling dynamic and anticipatory management of technological integration within clinical and organizational settings. Its multidimensional framework encompasses not only technical and economic dimensions but also ethical, social, regulatory, and organizational considerations, thereby providing a comprehensive basis for informed decision-making. In the field of omics sciences, CTA has notably been employed for evaluating technologies such as NGS applications in oncology (31), WGS in oncology (32–34), circulating tumor DNA (ctDNA) testing (35), and genomic profiling tools in the field of breast cancer (36;37). These are only a few examples of applications derived from the results of this survey, illustrating the application of CTA’s holistic and anticipatory assessment strategy, highlighting the framework’s strength in managing complex and rapidly evolving omics technologies. Despite these advantages, challenges persist in effectively integrating qualitative dimensions beyond cost-effectiveness analysis due to the absence of standardized methodologies across different processes of evidence generation. CTA aligns with VBHC principles by emphasizing early stakeholder involvement, patient perspectives, and ethical and organizational dimensions throughout the technology’s lifecycle. By adopting a dynamic and anticipatory approach, CTA supports equity of access and long-term system sustainability, ensuring that innovations are evaluated not only for clinical utility but also for their broader social and organizational implications. However, compared with more formalized frameworks, CTA relies less on standardized measurement of patient-reported outcomes or economic efficiency, which may limit comparability across settings.

## AETSA framework

The *Guide to procedures and methods for the assessment of omics technologies* (38) recently developed by the *Health Technology Assessment Area-AETSA, Andalusian Public Foundation Progress and Health-FPS*, for *REDETS* (39) in Spain, introduces a structured and specialized approach specifically tailored to omics technologies. The framework explicitly addresses key evaluation domains such as analytical validity, clinical validity, clinical utility, and notably, personal utility—an element uniquely emphasized due to the personalised nature of omics tests. It systematically guides the collection and assessment of evidence across multiple dimensions, including service delivery models and specific organizational aspects related to bioinformatics, recognizing the critical role of data analysis in omics applications. Additionally, the guide incorporates detailed criteria for summarizing collected evidence, focusing on net benefit, cost-effectiveness, feasibility, and encompassing ethical, legal, social, and cultural implications. A significant strength of this newly published and standardized framework lies in its flexibility, enabling adaptation to various omics contexts and its strong orientation toward patient-centered outcomes *and technological maturity assessment. Notably, this guide includes an abridged version intended to support the update of the Spanish catalogue of genetic and genomic tests within the common portfolio of services, enhancing its applicability to national health policy.* However, its comprehensiveness, though academically robust, has been perceived by stakeholders as potentially limiting practical usability due to excessive detail. This potential barrier is expected to be overcome once piloting begins using an abbreviated version that offers a more condensed and practical approach. The framework’s adaptability to different contexts is a key strength, but it has yet to undergo its first formal application in a real-world evaluation context. The AETSA framework explicitly incorporates VBHC elements by assessing analytical validity, clinical utility, and, distinctively, *personal utility*, thereby embedding patients’ goals and quality of life into the evaluation. It also addresses equity through allocative considerations and places strong emphasis on organizational feasibility and bioinformatics, linking system readiness with long-term sustainability. Ethical, legal, social, and cultural implications are systematically included, making this framework a comprehensive operationalization of the allocative, technical, personal, and societal dimensions of value in healthcare.

## Discussion

Our study analyzes the approach of different organizations working in the field of HTA toward the evaluation of omics technologies. Responses were obtained from thirty-nine organizations, of which twenty indicated performing assessments of omics technologies. Among these, only three organizations reported adopting a specific framework: the MSAC, CTA, and AETSA. Given the characteristics of the questionnaire, these three frameworks are the only ones analyzed in detail, as they were the only frameworks for which comprehensive information regarding their structure and application was collected.

The relatively small number of respondents that reported having assessed omics technologies or adopting a specific framework for their evaluation highlights an important gap in the current HTA landscape. Despite the growing relevance of omics in clinical and public health contexts, less than half of the surveyed organizations have engaged in their formal evaluation. This may be attributed to several factors. First, the technical complexity of omics tools can act as a barrier for agencies focused on traditional pharmaceutical products or medical devices (40;41). Additionally, the wide variety of omics approaches (e.g., genomic sequencing, multi-omics integration) introduces further challenges in standardizing assessment procedures (42). Limited prioritization by policymakers and resource constraints may also contribute to this gap, since decision-makers often focus HTA efforts on technologies with more immediate or population-level impact. Moreover, the substantial evidence and validation requirements for omics tools, including ethical considerations, represent additional barriers to producing clear, and actionable recommendations (43;44).

In this context, the frameworks reviewed in our study provide some useful directions for adapting HTA processes to better accommodate the particularities of omics technologies. Despite being applied in markedly different contexts, they all share a common emphasis on contextual factors as a core component of their evaluative approach. Unlike standard HTA approaches, which often emphasize internal validity, these frameworks underline contextual dimensions such as organizational readiness, ethical implications, and health system characteristics. This feature is particularly relevant for omics technologies, where the potential benefits and feasibility of implementation may vary significantly depending on the setting (45). Integrating personalized medicine into health systems requires careful attention to infrastructure, resources, ethical–legal–social implications, and education. Such comprehensive consideration ensures not only the technical feasibility but also the acceptance and sustainability of personalized medicine (46). For example, the organizational capacity to deliver care based on genomic information, the availability of trained personnel, and local economic, ethical, or legal constraints can all influence the success of omics-based interventions. The inclusion of such factors early in the assessment allows for a more practical evaluation of both the potential impact and the system’s ability to absorb and implement the innovation (47;48).

Another contact point among the analyzed frameworks is their openness to alternative forms of evidence generation. Omics technologies challenge traditional evidence hierarchies: they are often tailored to specific patient subgroups, involve complex diagnostic pathways, and may evolve rapidly, making RCTs less feasible or less informative (49). These frameworks acknowledge that evidence for omics may need to derive from a broader set of methodologies, such as observational studies, longitudinal cohorts, or real-world evidence. MSAC, for instance, promotes the use of linked evidence approaches that combine data from multiple sources across the care pathway. Similarly, CTA explicitly integrates mixed-method study designs and expert consensus in early assessment stages. AETSA, in turn, recognizes the importance of iterative evidence accumulation through implementation studies and stakeholder engagement. Importantly, this integration of diverse evidence sources does not imply a disregard for methodological rigor; rather, it highlights the need to critically appraise each type of evidence. This flexibility is particularly valuable when evaluating technologies still undergoing refinement or when clinical utility is context dependent (50).

The integration of VBHC principles further enhances the relevance of these frameworks. While all three approaches recognize the need to move beyond clinical efficacy, they do so in different ways. The MSAC framework balances technical and personal value through cost-utility analysis, explicit attention to equity, and the *“value of knowing,”* reflecting both individual and societal perspectives. CTA emphasizes early stakeholder engagement and ethical, organizational, and social considerations, aligning with the allocative and societal pillars of VBHC. The AETSA framework represents explicitly some VBHC principles by systematically including clinical and personal utility, thus encompassing the allocative, technical, personal, and societal dimensions of value (51). Taken together, these frameworks illustrate how VBHC can serve as a unifying perspective for the assessment of omics technologies. This perspective captures not only measurable outcomes but also quality of life, equity, and system sustainability, dimensions often crucial for innovations whose benefits emerge over the long term (51).

The findings of our study carry relevant implications for both policy-making and scientific research. At the scientific level, the analysis provides HTA organizations and institutions with an overview, albeit with acknowledged gaps, of international trends regarding the evaluation of omics technologies. This overview presents potential areas for reflection and future evaluative approaches. Importantly, our research highlights a significant gap in methodological tools specifically tailored to address the unique characteristics of omics technologies, underscoring a need for targeted research initiatives aimed at developing such methodologies. From a policy perspective, our work offers policymakers insights into diverse strategies available for approaching these HTA evaluations. Given the growing importance of HTA in guiding reimbursement decisions and clinical implementation, adopting methodological tools tailored specifically to omics is crucial for enabling informed decision-making. Strengthening collaboration between regulatory bodies, HTA institutions, and clinical stakeholders could support more consistent and timely evaluations. Future studies should investigate how these frameworks perform in real-world scenarios and examine their influence on decision-making outcomes. Comparative case studies and longitudinal follow-ups could yield valuable insights into the practical utility, adaptability, and impact of different HTA approaches.

It is also necessary to highlight some limitations of our study. First, some organizations may have developed or utilized omics-specific HTA frameworks not captured in our survey due to non-response or incomplete data. This limitation is inherent in voluntary, survey-based research, potentially affecting the representativeness of our results. Future research could address this gap by combining survey methodologies with targeted follow-up interviews or systematic reviews of grey literature and institutional reports. Additionally, despite 20 organizations reporting evaluations of omics technologies, the survey produced limited qualitative or structured information, especially among respondents not utilizing specific frameworks. This limitation reduced the depth of comparison and prevented further exploration of institutional practices. The heterogeneity among the frameworks considered also limits direct comparisons: varying maturity levels and application across different health systems challenge the generalizability of the findings. Moreover, although the AETSA framework is specifically designed for omics technologies, it remains theoretical, with its real-world applicability yet to be validated. Furthermore, it is important to contextualize these three frameworks within a wider methodological landscape. Established instruments such as the *EUnetHTA HTA Core Model* have already codified multidimensional approaches to value assessment across nine domains, including clinical, economic, organizational, ethical, and social aspects (52). Placing MSAC, CTA, and AETSA in this broader context shows how national and regional adaptations complement international standards and highlights the need for comparative research linking omics-specific frameworks with established tools (52).

Beyond the challenges observed in high-income settings, it is also important to reflect on how these frameworks could be adapted and applied in resource-constrained environments. In particular, the experience of low- and middle-income countries (LMICs) offers valuable insights into how HTA approaches may evolve under limited financial and technical capacity. In these environments, the comprehensive structure of the frameworks may require adaptation to local priorities, data availability, and institutional capacity (53). Experiences from international initiatives demonstrate that simplified, context-sensitive HTA approaches can effectively support priority setting and universal health coverage policies, even when financial and technical resources are limited (54). In this perspective, value-based frameworks could serve as flexible templates: by emphasizing allocative efficiency, stakeholder participation, and transparency, they can guide incremental capacity-building for the assessment of emerging technologies, including omics tools.

## Conclusions

Omics technologies offer promising opportunities for advancing personalized and precision medicine, yet their adoption in clinical practice remains limited due to challenges in generating robust and context-sensitive evidence. This study explored how organizations working in the HTA field are addressing these challenges. While nearly half of the respondents reported experience in evaluating omics technologies, only a few have adopted specific frameworks for their assessment. The analysis of the MSAC, CTA, and AETSA frameworks highlights useful elements, such as attention to context, flexible evidence requirements, and value-based healthcare principles, that align well with the specific nature of omics technologies. These features can help overcome some of the limitations of traditional HTA approaches when applied to fast-evolving, personalized interventions. Nonetheless, further methodological refinement and institutional investment are needed. Developing effective HTA strategies for omics requires balancing standardization with adaptability and ensuring organizations are equipped to address the complexity and multidisciplinary nature of these technologies. Future research could advance these findings through (i) systematic reviews of grey literature and institutional reports to capture additional frameworks; (ii) in-depth case studies of countries currently piloting omics-specific HTA approaches; and (iii) longitudinal assessments to evaluate real-world feasibility, sustainability, and impact. Moreover, particular attention should be given to how these frameworks can be adapted for use in LMICs, where simplified, resource-sensitive approaches are essential to ensure equity and sustainability in the evaluation and adoption of omics technologies.

## Supporting information

Osti et al. supplementary material Osti et al. supplementary material

## Supplementary material

The supplementary material for this article can be found at http://doi.org/10.1017/S0266462325103279.
