# Supplementary material for: Value-based health care frameworks for the health technology assessments of “omics” technologies: an international survey
Source: Int J Technol Assess Health Care. 2025 Nov 28;41(1):e80. doi: 10.1017/S0266462325103279 (PMC12689241; doi:10.1017/S0266462325103279)
Supplement: Osti et al. supplementary material [file S0266462325103279sup001.pdf]

HTA Frameworks on the evaluation of genomic technologies: a survey to experts

Background

In recent decades, medicine has witnessed a real revolution in genetics and omics sciences; the understanding of the role of the human genome in the onset of diseases and their potential prevention is becoming more and more refined, and with it the prospects in integrating these notions into clinical practice and public health strategies in order to promote the concept of personalized medicine.[1] Despite excellent premises, discoveries in the field of genomics have so far struggled to translate into innovation from the perspective of clinical practice, often getting bogged down in implementation issues ranging from ethical, social, and legal problems to the availability of data in terms of clinical efficacy, or financial cover for their sustainable introduction into a health care system. [2] For these reasons, the role of HTA in the evaluation of genomic technologies assumes central importance, identifying itself as a preferred tool for providing answers to many of the gaps and bottlenecks of implementations in this field. In analyzing, however, the application of classical HTA vis-à-vis omics technologies, potential difficulties related to the diversity and peculiarities of data concerning this area and feeding into related frameworks are highlighted.[3]

To shed light on the views of the international scientific community regarding the application of HTA Frameworks used in the context of omics technologies, this brief survey was developed as part of the ExACT project by the Basque Office for Health Technology Assessment (OSTEBA) and Università Cattolica del Sacro Cuore (UCSC).

The ExACT project

The "European network staff eXchange for integrAting precision health in the health Care sysTems" consortium (ExACT), is aimed at building a community of academic and non-academic institutions that generates high quality, multidisciplinary collaboration by exchanging knowledge in research and training activities on precision health. Precision health aims to prevent and predict illness, maintaining health and quality of life for as long as possible, by drawing on the new technological and data science tools to translate volumes of research and clinical data into information that citizens, patients and doctors can use. The implementation of precision health remains contingent on significant data

The ExACT project has received funding from the European Union's Horizon 2020 research and innovation programme under grant agreement No 823995

acquisition and timely analysis to determine the most appropriate basis on which to tailor health optimization for individual in the prevention, diagnosis, and disease treatment. Achieving effective and proportionate governance of health-related data will be essential for the future health care systems.

The ExACT consortium consists of 15 international Partners and is coordinated by the Catholic University of the Sacred Heart. More information on the project website http://www.exactproject.net/site/index.php. The ExACT project has received funding from the European Union's Horizon 2020 research and innovation programme MSCA-RISE-2017 Marie Skłodowska-Curie Research and Innovation Staff Exchange (RISE) under the grant agreement 823995.

Questions

1. **Please indicate the name of your agency/organization.**
2. **What kind of health technologies are of interest for your agency's activity. Could you provide us some examples?**

   Pharmaceuticals

   Devices

   Diagnostics

   Surgical interventions

   Medical procedures

   Hospital care

   Community care/ programmes

   Public health interventions
3. **Does your agency/organization perform HTA assessment of omics technologies (genomics, transcriptomics, proteomics, metabolomics)?**

   Yes

   No
4. **If your answer is yes, please indicate the omics technologies your agency/organization has performed an HTA of and indicate a link to the report.**
5. **If your answer is yes, does your agency/organization use a specific evaluation framework for an HTA of omics technologies?**

6. **If your answer is yes, please indicate the framework used.**

Risk-Benefit Framework

ACCE

EGGAP

Practical framework Stages of translational research

HTA core model

Framework for the Evaluation of Measures of Genomic Diagnostics

Genome-based Knowledge Management in Cycles model (G-KNOMIC)

SynFRAME

A novel framework for stakeholder- informed prioritization of cancer genomics research

Completeness framework

New framework

PHG New framework

Framework for the Assessment of Genetic Testing in the Andalusian Public Health System

GETT: a Genetic testing Evidence Tracking Tool

Ontario advisory committee on coverage decisions for new predictive genetic tests

INESSS Framework

Fryback- Thornbury Evaluation Framework

The United States Preventive Services Task Force (USPSTF) Evaluation Model

ACHDNC analytic framework

Rapid ACCE

EGGAP update

UKGTN Gene Dossier

Other (indicate)

The ExACT project has received funding from the European Union's Horizon 2020 research and innovation programme under grant agreement No 823995

7. **What are in your opinion the main strengths of the Framework you adopt? (e.g. presence of specific questions related to the technology being evaluated, ease in retrieving data related to the domains being analyzed, easy interpretability, adaptability to different technologies in the friend...)**
8. **What are, in your opinion, the main limitations of the framework you adopt? (by "limitations" we intend something that is strictly related to the nature of the framework itself e.g. poor adaptability of the framework to omics technologies, insufficient number of fields to present a complete overview of the technology, lack of attention to the context-related evaluation components…)**
9. **Could you identify some gaps and barriers into the application of this framework? (by "barriers" we intend something that is related to the context the framework is applied to e.g. insufficient quality of the available evidence, lack of timeliness of research, lack of contact and interaction among policymakers/researchers/stakeholders, lack of standardization in the usage of the framework…)**
10. **Please, if not mentioned, indicate other HTA frameworks used for omics technology evaluation that you are aware of.**

The ExACT project has received funding from the European Union's Horizon 2020 research and innovation programme under grant agreement No 823995

References

1. Boccia S. Why is personalized medicine relevant to public health? The European Journal of Public Health. 2014;24:349–50.

2. Klein ME, Parvez MM, Shin J-G. Clinical Implementation of Pharmacogenomics for Personalized Precision Medicine: Barriers and Solutions. J Pharm Sci. 2017;106:2368–79.

3. Becla L, Lunshof JE, Gurwitz D, Schulte in den Bäumen T, Westerhoff H V., Lange BMH, et al. Health technology assessment in the era of personalized health care. Int J Technol Assess Health Care. 2011;27:118–26.

The ExACT project has received funding from the European Union's Horizon 2020 research and innovation programme under grant agreement No 823995
